# Multi-ethnic genome-wide association study identifies novel locus for type 2 diabetes susceptibility

**DOI:** 10.1038/ejhg.2016.17

**Published:** 2016-05-18

**Authors:** James P Cook, Andrew P Morris

**Affiliations:** 1 Department of Biostatistics, University of Liverpool, Liverpool, UK; 2 Wellcome Trust Centre for Human Genetics, University of Oxford, Oxford, UK

## Abstract

Genome-wide association studies (GWAS) have traditionally been undertaken in homogeneous populations from the same ancestry group. However, with the increasing availability of GWAS in large-scale multi-ethnic cohorts, we have evaluated a framework for detecting association of genetic variants with complex traits, allowing for population structure, and developed a powerful test of heterogeneity in allelic effects between ancestry groups. We have applied the methodology to identify and characterise loci associated with susceptibility to type 2 diabetes (T2D) using GWAS data from the Resource for Genetic Epidemiology on Adult Health and Aging, a large multi-ethnic population-based cohort, created for investigating the genetic and environmental basis of age-related diseases. We identified a novel locus for T2D susceptibility at genome-wide significance (*P*<5 × 10^−8^) that maps to *TOMM40-APOE*, a region previously implicated in lipid metabolism and Alzheimer's disease. We have also confirmed previous reports that single-nucleotide polymorphisms at the *TCF7L2* locus demonstrate the greatest extent of heterogeneity in allelic effects between ethnic groups, with the lowest risk observed in populations of East Asian ancestry.

## Introduction

Genome-wide association studies (GWAS) of complex human traits have traditionally been undertaken in homogeneous populations from the same ancestry group because: (i) geographical confounding between the trait and genetic variation can inflate type I error rates, if not accounted for in the association analysis;^1^ and (ii) there may be reduced power to detect association due to heterogeneity in allelic effects on the trait between ethnicities.^2^ However, more recent GWAS have been performed in large multi-ethnic cohorts,^3, 4^ where assignment of individuals to homogeneous population groups becomes increasingly difficult because of ancestral diversity and admixture.

Methodology to detect and adjust for confounding between a trait and genetic variation is well established. A common approach is to apply principal components analysis (PCA) to a genetic relatedness matrix (GRM) between individuals. The first eigenvectors of the GRM represent linear combinations of single-nucleotide polymorphisms (SNPs), or axes of genetic variation (AGV), that best distinguish genetically dissimilar individuals, such as those of diverse ancestry. For example, application of PCA to reference samples from the International HapMap Project^5^ or the 1000 Genomes Project^6^ have been shown to generate two AGV that distinguish individuals of African, European and East Asian ancestry.^7^ Within ancestry groups, PCA has also been demonstrated as a powerful tool to infer structure at a finer scale,^8, 9^ and the AGV can be included as covariates in association analyses to account for geographical confounding with the trait.^10^

With the increasing availability of GWAS data from diverse populations, trans-ethnic meta-analysis of association summary statistics across ancestry groups may offer increased power to detect novel loci for complex traits through increased sample size.^11^ There is increasing evidence from these studies that common variant association signals for complex traits are, in fact, shared across ancestry groups, and there is relatively little heterogeneity in allelic effects between populations.^12^ Furthermore, methodology to account for any such heterogeneity is well developed,^2^ and the differential structure of linkage disequilibrium (LD) between ancestries may, in fact, be beneficial for localising causal variants in trans-ethnic fine-mapping studies.^13^

In this article, we have developed and evaluated an approach for the analysis of GWAS of complex human traits in a multi-ethnic cohort to: (i) test for association whilst accounting for confounding with population structure by adjusting for AGV; and (ii) test for heterogeneity in allelic effects between ethnicities by considering an interaction with the first two AGV that distinguish broad ancestry groups. We have also applied this approach to the detection of loci associated with type 2 diabetes (T2D), and characterisation of the effects of lead SNPs across ancestry groups, through application to GWAS data from the multi-ethnic Resource for Genetic Epidemiology on Adult Health and Aging (GERA) cohort (database of Genotypes and Phenotypes (dbGaP) phs000674.p1).

## Materials and methods

### Multi-ethnic test of association in generalised linear modelling framework

Consider a sample of unrelated individuals from a multi-ethnic cohort, with phenotypes and genome-wide genotypes denoted by **y** and **G**, respectively. To account for population structure, within and between ethnicities, we considered AGV, denoted **x**, that were constructed through PCA of the GRM obtained from **G**. To test for association of the *j*th variant with phenotype, we considered a generalised linear modelling framework, given by


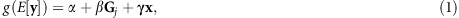


where *g*(.) is the link function. In this expression, *β* is the effect of the *j*th variant on phenotype, with genotypes coded under an additive model, and **γ** is a vector of regression coefficients for the AGV. A likelihood ratio test with one degree of freedom was then formed by comparing the maximised log-likelihood of the unconstrained model (1), with that obtained under the null hypothesis of no association, *β*=0.

### Test of heterogeneity in allelic effects between ethnicities

To test for heterogeneity of allelic effects between ethnicities at the *j*th SNP, we extended model (1) to include an interaction between genotype and the first two AGV, **x**_1_ and **x**_2_, which we expect to distinguish between individuals from diverse ethnicities, given by


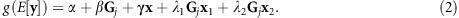


In this expression, *λ*_1_ and *λ*_2_ are regression coefficients for the interaction between the first two AGV and genotypes at the *j*th variant. A likelihood ratio test with two degrees of freedom was then formed by comparing the maximised log-likelihood of the unconstrained model (2) with that obtained under the null hypothesis of no heterogeneity, *λ*_1_=*λ*_2_=0.

### Simulation study

We performed a detailed simulation study to investigate the type I error rate and power of the generalised linear modelling approach, with and without adjustment for AGV, to detect: (i) association between a variant and a case-control phenotype; and (ii) heterogeneity of allelic effects on the phenotype at the variant. We considered the 10 reference populations from Phase 3 of the International HapMap Project,^5^ incorporating haplotypes of African, East Asian, South Asian, Hispanic and European ancestry, to simulate a multi-ethnic cohort of 20 000 individuals. Five of the reference populations (ASW, MKK, LWK, MXL and GIH) are admixed, in which individuals have varying continental ancestry proportions, whilst the remainder (CEU, TSI, YRI, CHB/JPT, and CHD) are relatively homogeneous.^5^ We began by generating genome-wide genotype data for 2000 individuals from each of the reference populations using HAPGENv2.^14^ We constructed a GRM from pair-wise identity by descent metrics estimated from LD pruned (*r*^2^<0.01 across individuals) autosomal SNPs with MAF⩾1%. We applied PCA to the GRM to obtain: (i) 10 ‘multi-ethnic' AGV to account for structure between and within ancestry groups; and (ii) four ‘population-specific' AGV to account for structure within each population.

We considered a range of models of association of a causal SNP with the dichotomous phenotype across ethnic groups, parameterised in terms of the allelic effect (log-odds ratio) of the alternative allele in each population (Supplementary Table S1). These models incorporate heterogeneity in allelic effect sizes between ethnic groups: (a) African-specific effect; (b) African vs others; and (c) East Asian vs European, South Asian and Hispanic. In model (a), the allelic effect of the causal SNP is specific to the four African ancestry populations (MKK, ASW, LWK and YRI). In model (b), the causal SNP has opposing allelic effects, of the same magnitude, in African ancestry populations versus all others. In model (c), the causal SNP has opposing allelic effects, of the same magnitude in East Asian ancestry populations (CHB/JPT and CHD) versus European, South Asian and Hispanic ancestry populations (GIH, MXL, CEU and TSI), but no effect in African ancestry populations. We also introduced trans-ethnic structure (moderate and extreme) by varying the ratio of cases to controls in each population, whilst fixing the total sample size, thereby inducing confounding between the phenotype and ancestry group (Supplementary Table S2).

For each scenario, we generated 1000 replicates of genotype data for the causal SNP for the multi-ethnic cohort of 20 000 individuals. For each replicate, we selected the causal SNP at random from those present in the Phase 3 HapMap reference panel,^5^ and obtained the alternative allele frequency in each population. Genotypes were then simulated in the required number of cases and controls in each population, according to the population-specific allele frequency and odds ratio. For each replicate of data, we tested for association between the causal SNP and phenotype: (i) with no correction for population structure; and (ii) by inclusion of the 10 ‘multi-ethnic' AGV as covariates in the logistic regression model. We also tested for heterogeneity in allelic effects of the causal SNP across ethnicities by including an interaction with the first two AGV as additional covariates. For comparison, we tested for association between the causal SNP and phenotype within each of the 10 populations, separately, by inclusion of the four ‘population-specific' AGV as covariates in the logistic regression model. We combined association summary statistics across populations through fixed-effects meta-analysis with inverse-variance weighting of effect sizes. We also assessed evidence for heterogeneity in allelic effects between populations in the meta-analysis by means of Cochran's *Q* statistic. Each test of association and/or heterogeneity was evaluated at nominal significance thresholds of *P*<0.05 and *P*<0.01, and at the traditional genome-wide standard of *P*<5 × 10^−8^.

### GWAS of T2D susceptibility in GERA

We applied the methodology developed and evaluated in this article to identify and characterise loci associated with T2D susceptibility using GWAS data from GERA, a large multi-ethnic population-based cohort, created for investigating the genetic and environmental basis of age-related diseases (dbGaP phs000674.p1). T2D status is based on ICD-9 codes in linked electronic medical health records. Participants in the GERA cohort have previously been genotyped using one of four custom arrays, which have been designed to maximise coverage of common and low-frequency variants in non-Hispanic white, East Asian, African American and Latino ethnicities.^15, 16^ We undertook quality control of these genotype data, removing individuals from known pedigrees and/or with call rate (<97%), and excluding SNPs on the basis of call rate (<95%) and extreme deviation from Hardy–Weinberg equilibrium (autosomes only, exact *P*<10^−6^). We constructed a GRM from pair-wise identity by descent metrics estimated from LD pruned (*r*^2^<0.01 across individuals) autosomal SNPs shared across the four genotyping arrays, and with MAF⩾1%, after exclusion of those in high LD and complex regions, and those mapping to established T2D loci. We defined related individuals on the basis of pi-hat>0.2, and removed those from each family set with the lowest call rate.

We applied PCA to the GRM to obtain AGV, the first 20 of which were tested for association with T2D in a logistic regression model to determine confounding with disease status. AGV correlated with T2D at nominal significance (*P*<0.05) were retained as covariates in downstream SNP association analyses.

SNPs passing initial quality control were lifted to NCBI build GRCh37 (UCSC hg19 assembly) of the human genome, and were excluded if they then had unknown position. SNPs were then removed if alleles did not match those reported in the 1000 Genomes Project multi-ethnic reference panel^6^ (autosomes, phase 3, October 2014 release; X chromosome, phase 1, March 2012 release) or if they were palindromic (AT/GC) to avoid strand errors. For each of the four genotyping arrays separately, we constructed a scaffold for imputation after excluding SNPs with MAF<1%, which was then pre-phased using SHAPEITv2.5.^17^ The resulting haplotypes were imputed, separately for each genotyping array, up to the 1000 Genomes Project reference panel (autosomes, phase 3, October 2014 release; X chromosome, phase 1, March 2012 release) using IMPUTEv2.3.^18^ Data were then merged across the four genotyping arrays, and SNPs excluded from downstream association analyses if rare and/or poorly imputed (MAF<0.5%, IMPUTE info<0.4).

We used SNPTESTv2.5^19^ to test for association of T2D with each SNP in a logistic regression framework under a dosage model, and after adjustment for sex and AGV (described above) as covariates to take account of trans-ethnic and ancestry-specific population structure. The genomic control inflation factor^20^ *λ*_1000_ was calculated to assess the evidence for residual population structure that is not accounted for by covariate adjustment. Genome-wide significance was defined by the traditional threshold of *P*<5 × 10^−8^. We tested for heterogeneity in allelic effects on T2D between ancestry groups by including an interaction between genotypes and the first two AGV from PCA in the logistic regression model.

## Results

### Simulation study

Under the null hypothesis of no association with the phenotype, type I error rates were substantially inflated in the presence of population structure (moderate or extreme) without adjustment for AGV, as expected (Supplementary Figure S1, Supplementary Table S3). However, type I error rates were well controlled by inclusion of 10 ‘multi-ethnic' AGV as covariates in the generalised linear regression model, even in the presence of extreme population structure. Type I error rates were also well controlled by testing for association within each homogenous population, adjusting for four ‘population-specific' AGV, and combining summary statistics via fixed-effects meta-analysis with inverse-variance weighting of effect sizes.

**Figure 1:**
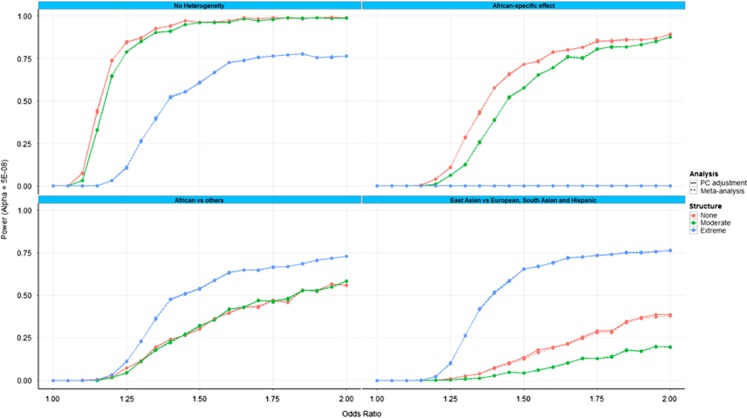
Power to detect association (at genome-wide significance, *P*<5 × 10^−8^) as a function of the allelic effect size for: (i) the logistic regression model after adjustment for 10 AGV as covariates; and (ii) fixed-effects meta-analysis of summary statistics across populations via inverse-variance weighting of effect sizes. The four panels correspond to alternative models of heterogeneity of allelic effects between ancestry groups (defined in Supplementary Table S1). Within each panel, results are presented for extreme, moderate and no population structure (defined in Supplementary Table S2).

Figure 1 presents the power to detect association (at genome-wide significance, *P*<5 × 10^−8^) with the causal variant as a function of the allelic effect size: (i) for the generalised linear model after adjustment for 10 ‘multi-ethnic' AGV as covariates for all populations combined; and (ii) for fixed-effects meta-analysis across populations with inverse-variance weighting of effect sizes, obtained after adjusting for four ‘population-specific' AGV. The four panels correspond to alternative models of heterogeneity of allelic effects between ancestry groups (defined in Supplementary Table S1). Within each panel, results are presented for extreme, moderate and no population structure (defined in Supplementary Table S2). Power for the generalised linear regression model and meta-analysis is indistinguishable in all scenarios.

In the absence of heterogeneity in allelic effects between ancestry groups, power decreases with the extent of population structure, as expected, because variation in phenotype can be increasingly explained by ethnicity. A similar pattern of results is observed for the model of heterogeneity incorporating an African-specific allelic effect when there is no or moderate population structure. However, under extreme population structure, there is no power to detect association with an African-specific allelic effect because cases are present only in populations of non-African ancestry. For the remaining two models of heterogeneity in allelic effects between ancestry groups, power to detect association is greatest in the presence of extreme population structure. This pattern of results does not reflect inflated type I error rates under extreme population structure, as described above (Supplementary Figure S1), but corresponds to configurations of case:control ratios in populations that mimic the direction of effect of the causal variant (that is, reduced prevalence in populations in which the allele is protective).

**Figure 2:**
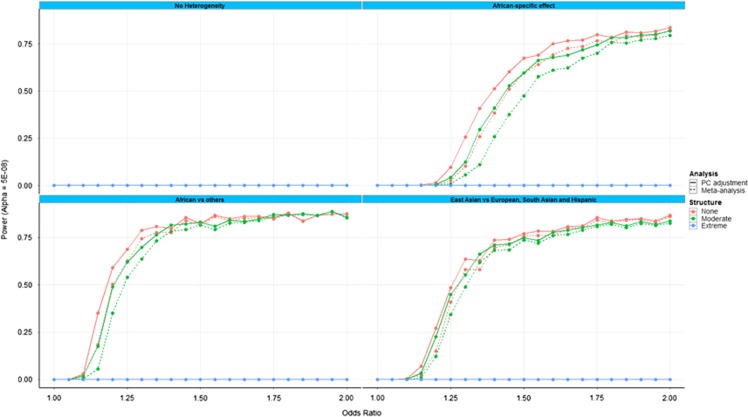
Power to detect heterogeneity in allelic effects between ancestry groups (at genome-wide significance, *P*<5 × 10^−8^), as a function of allelic effect size for: (i) the logistic regression model by including interactions of the variant with the first two AGV (and with adjustment for the first 10 as covariates); and (ii) Cochran's *Q* statistic from fixed-effects meta-analysis of summary statistics across populations via inverse-variance weighting of effect sizes. The four panels correspond to alternative models of heterogeneity of allelic effects between ancestry groups (defined in Supplementary Table S1). Within each panel, results are presented for extreme, moderate and no population structure (defined in Supplementary Table S2).

Figure 2 presents the power to detect heterogeneity in allelic effects between ancestry groups (at genome-wide significance, *P*<5 × 10^−8^), as a function of allelic effect size, assessed by: (i) including interactions of the variant with the first two AGV in the generalised linear regression model; and (ii) Cochran's *Q* statistic in the fixed-effects meta-analysis. The four panels correspond to alternative models of heterogeneity of allelic effects between ancestry groups (defined in Supplementary Table S1). Within each panel, results are presented for extreme, moderate and no population structure (defined in Supplementary Table 2). As expected, under a model of homogenous allelic effects across populations, there is no power to detect heterogeneity. Furthermore, in the presence of extreme population structure, there was little power to detect heterogeneity under any model because of the strong confounding of disease status and ancestry. Overall, power to detect heterogeneity was greater for the interaction in the generalised linear regression model than for Cochran's *Q* statistic in the meta-analysis, reflecting the reduced numbers of degrees of freedom in the test.

### GWAS of T2D susceptibility in GERA

After quality control, a total of 71 604 unrelated participants, including 9747 T2D cases, were retained for analysis. The majority of GERA participants (79.3%) were self-reported non-Hispanic white, although there was considerable variability in ethnicity according to genotyping array (Supplementary Table S4, Supplementary Figure S2). As expected, the first two AGV from PCA broadly differentiate individuals of African, European and East Asian ancestry (Supplementary Figure S2). A total of nine AGV were correlated with T2D at nominal significance (*P*<0.05), and were retained as covariates in downstream SNP association analyses (Supplementary Table S5, Supplementary Figure S3). The genomic control inflation factor, *λ*_1000_=1.005, indicated no evidence of residual structure that is not accounted for by this covariate adjustment (Supplementary Figure S4). Variants at 10 loci attained genome-wide significant evidence of association with T2D (Table 1, Supplementary Figure S4), with the strongest signals mapping to *TCF7L2* (lead SNP rs34872471, NC_000010.10:g.114754071T>C, *P*=6.4 × 10^−53^) and *IGF2BP2* (lead SNP rs11927381, NC_000003.11:g.185508591T>C, *P*=3.0 × 10^−14^).

Amongst the loci attaining genome-wide significance was a novel association signal for T2D susceptibility, mapping to *TOMM40-APOE* (lead SNP rs157582, NC_000019.9:g.45396219C>T, *P*=2.8 × 10^−9^). This association signal maps ~800 kb upstream of the previously reported locus for T2D susceptibility at *GIPR*^21^ (Supplementary Figure S5). However, through conditional analysis, by including genotypes at the lead SNP at the *GIPR* locus, rs200706727 (NC_000019.9:g.46157838_46157839insG), as an additional covariate in the logistic regression model, the novel association signal at *TOMM40-APOE* was not attenuated (*p*_COND_=3.2 × 10^−9^), and thus demonstrated to be distinct.

We tested for heterogeneity in allelic effects on T2D susceptibility between ancestry groups for lead SNPs at the 10 loci attaining genome-wide significant evidence of association in the multi-ethnic analysis. We observed nominal evidence of interaction (*p*_INT_<0.05) only at the lead SNP, rs34872471, at the *TCF7L2* locus (*p*_INT_=0.012). Specifically, the odds ratio at the SNP increased with larger values of the first AGV, which corresponds to a smaller effect on T2D susceptibility in individuals of East Asian ancestry (Supplementary Figure S2, Supplementary Table S6).

## Discussion

In this article, we have evaluated a framework for detecting association of genetic variants with a complex trait in multi-ethnic cohorts, allowing for population structure, and developed a powerful test of heterogeneity in allelic effects between ancestry groups. Through simulation, we have demonstrated that adjustment for AGV as covariates in a generalised linear regression modelling framework can control type I error rates for association testing in multi-ethnic cohorts. By additionally including interaction between a SNP and the first two AGV in the model, we achieve greater power to detect heterogeneity in allelic effects between ancestry groups than by assessment of Cochran's *Q* statistic in a fixed-effects meta-analysis of homogeneous population groups.

Encouraged by the results of our simulation study, we applied the methodology developed and evaluated in this article to identify and characterise loci associated with T2D susceptibility using GWAS data from a large multi-ethnic population-based cohort. Amongst the loci attaining genome-wide significance in our multi-ethnic analysis was a novel association signal for T2D susceptibility, mapping to *TOMM40-APOE*. Our lead SNP has not been interrogated in previous GWAS meta-analyses of T2D susceptibility because it is not present on widely used genotyping arrays or previous reference panels from the International HapMap Project and phase 1 of the 1000 Genomes Project. The closest proxy for our lead SNP in HapMap is rs6857 (NC_000019.9:g.45392254C>T, EUR *r*^2^=0.758 with rs157582) and attains nominal T2D association in GERA (*P*=1.6 × 10^−6^). The association signal for this SNP was replicated in meta-analyses of T2D GWAS of up to 12 171 cases and 56 862 controls of European ancestry from the DIAGRAM Consortium^21^ (*P*=0.0025). There was no evidence of heterogeneity in allelic effects between GERA and DIAGRAM (Cochran's *Q P*=0.95), with consistent odds ratios observed in both studies (Supplementary Table S7). Common SNPs mapping to the *APOE* locus have previously been associated with low- and high-density lipoprotein and total cholesterol,^22^ although the reported lead SNP for these lipids traits (rs4420638, NC_000019.9:g.45422946A>G) is only in moderate LD with that which we report here for T2D susceptibility (EUR *r*^2^=0.334 with rs157582).

Variation mapping to the *TOMM40-APOE* locus has also previously been associated with late onset Alzheimer's disease (LOAD). Compared with the common *APOE* ɛ3 allele, ɛ4 increases the risk of LOAD and lowers the age at onset of AD in a dose-dependent fashion, whereas the ɛ2 allele confers a protective effect.^23, 24^ *APOE* alleles are tagged by two SNPs in the region, rs429358 (NC_000019.9:g.45411941T>C) and rs7412 (NC_000019.9:g.45412079C>T), which are not present on the majority of commercial genome-wide genotyping arrays, but can be well imputed using reference panels from the 1000 Genomes Project.^25^ In particular, rs429358, which defines the ɛ4 LOAD risk allele, is in strong LD with our lead SNP (EUR *r*^2^=0.524 with rs157582), and can consequently partially explain the T2D association signal in conditional analyses (Supplementary Table S8). Given that T2D also occurs later in life, and that the effect of *APOE* ɛ4 on Alzheimer's disease is large (odds ratio of 2.84 per allele^23^), we were unclear whether our observed diabetes association at this locus might thus reflect under-representation of LOAD patients amongst GERA cases and confounding with age. To investigate the influence of this potential ascertainment bias, we considered the impact of age on the allelic effect of rs157582 on T2D susceptibility. We observed no evidence of an interaction (*P*=0.83) between age and the SNP, after adjusting for the main effects of both variables in the logistic regression model. We also observed consistent allelic effects of rs157582 on T2D susceptibility after stratifying participants according to their year of birth (Supplementary Table S9). Taken together, these data suggest that the T2D association at the *TOMM40-APOE* locus cannot be fully explained by LOAD ascertainment bias. However, to fully disentangle the association signals for T2D and LOAD at this locus would require case-control status for both diseases in the same sample of individuals, which is beyond the scope of this study.

Large-scale trans-ethnic meta-analyses have also demonstrated that many T2D GWAS loci are shared across diverse populations and that the allelic effects of lead SNPs at common variant association signals are predominantly homogeneous across ancestry groups.^26^ Amongst lead SNPs at loci attaining genome-wide significance in our multi-ethnic analysis, we detected nominal evidence of interaction with the first two AGV only at *TCF7L2*, where a smaller allelic effect on T2D susceptibility in individuals of East Asian ancestry was observed than in other ethnic groups. We recognise that the odds ratios at established T2D susceptibility loci are towards the lower end of those considered in our simulation study, and thus that power to detect heterogeneity in allelic effects between populations is limited. However, our results are consistent with previous findings from trans-ethnic meta-analysis of T2D susceptibility GWAS that highlighted heterogeneous allelic effects between ancestry groups at *TCF7L2*, where the smallest odds ratios for the disease were observed in populations of East Asian descent.^26^

The choice of analysis strategy in multi-ethnic GWAS will often be restricted by study design. When aggregating individuals from diverse populations from distinct GWAS, it may be impractical to exchange individual level genotype data, and trans-ethnic meta-analysis will be the only practical approach. On the other hand, in large, multi-ethnic GWAS, which include individuals of admixed descent, such as GERA, it will likely be difficult to define homogenous population groups for ethnic-specific analyses, and self-reported ancestry may not always be reliable. Our results would indicate that power to detect association is equivalent through meta-analysis of GWAS undertaken in homogeneous ancestry groups or multi-ethnic studies with adjustment for population structure. However, we have demonstrated increased power to detect heterogeneity in allelic effects between diverse populations in a combined multi-ethnic GWAS that incorporates interaction with AGV that distinguish major ancestry groups.

Specialist meta-analysis approaches for aggregating association summary statistics from multi-ethnic GWAS have also been developed, including MANTRA.^2^ This Bayesian ‘hybrid' of fixed- and random-effects meta-analysis allows for heterogeneity in allelic effects between populations according to a model of relatedness between them. In principal, the multi-ethnic GWAS analysis proposed here could be extended to a ‘joint test' of the main effect of the SNP and the interaction with the first two AGV, thereby allowing for heterogeneity in allelic odds ratios between diverse populations in evaluating the evidence of association with the trait. As with MANTRA, this approach would be particularly applicable in the context of trans-ethnic fine-mapping, where heterogeneity in allelic effects is driven by differential patterns of LD of SNPs with the causal variant in diverse populations, but is leveraged to localise the association signal.

In summary, through multi-ethnic association analysis in the GERA cohort, we have identified a novel locus for T2D susceptibility mapping to *TOMM40-APOE*, a region previously implicated in lipid metabolism and Alzheimer's disease. We have also confirmed previous reports of heterogeneity in allelic effects at the *TCF7L2* locus, where the lead SNP demonstrates the lowest risk for T2D in populations of East Asian ancestry. Further research will be required to determine the cause of this heterogeneity, but allelic effect size differences could reflect interaction of the causal variant with environmental risk factors that differ in exposure between ethnicities. With the increasing availability of large-scale multi-ethnic cohorts, our results will also help to inform future GWAS design and application of methodologies for complex trait locus discovery and characterisation.

## Figures and Tables

**Table 1:** Association summary statistics for T2D for lead SNPs attaining genome-wide significance (*P*<5 × 10^−8^) in 9747 cases and 61 857 controls from the GERA cohort

					*Alleles*					
*Locus*	*Lead SNP*	*Chr*	*Position*^a^ *(bp)*	*HGVS ID*	*Risk*	*Other*	*RAF*	P*-value*	*OR (95% CI)*	*Info*
*TCF7L2*	rs34872471	10	114 754 071	NC_000010.10:g.114754071T>C	C	T	0.280	6.4 × 10^−53^	1.31 (1.26−1.35)	0.963
*IGF2BP2*	rs11927381	3	185 508 591	NC_000003.11:g.185508591T>C	C	T	0.325	3.0 × 10^−14^	1.14 (1.10−1.17)	0.999
*JAZF1*	rs849134	7	28 196 222	NC_000007.13:g.28196222A>G	A	G	0.531	6.4 × 10^−13^	1.12 (1.09–1.16)	1.000
*SLC30A8*	rs13266634	8	118 184 783	NC_000008.10:g.118184783C>T	C	T	0.695	1.4 × 10^−11^	1.12 (1.09–1.16)	0.999
*CDKAL1*	rs7766070	6	20 686 573	NC_000006.11:g.20686573C>A	A	C	0.274	1.9 × 10^−11^	1.12 (1.09–1.16)	0.999
*CDKN2A-B*	rs10811661	9	22 134 094	NC_000009.11:g.22134094T>C	T	C	0.815	2.3 × 10^−10^	1.14 (1.09–1.19)	1.000
*MHC*	rs9273401	6	32 627 129	NC_000006.11:g.32627129A>G	G	A	0.112	1.8 × 10^−9^	1.16 (1.11–1.22)	0.926
*MACF1*	rs3768321	1	40 035 928	NC_000001.10:g.40035928G>T	T	G	0.185	2.4 × 10^−9^	1.13 (1.08–1.17)	0.984
*TOMM40-APOE*	rs157582	19	45 396 219	NC_000019.9:g.45396219C>T	C	T	0.766	2.8 × 10^−9^	1.13 (1.08–1.17)	0.858
*ANKRD55*	rs9687833	5	55 861 601	NC_000005.9:g.55861601G>A	A	G	0.200	2.9 × 10^−9^	1.12 (1.08–1.16)	1.000

Abbreviations: Chr, chromosome; CI, confidence interval; GERA, Genetic Epidemiology on Adult Health and Aging; OR, odds ratio; RAF, risk allele frequency; SNPs, single-nucleotide polymorphisms; T2D, type 2 diabetes.

a Position reported for NCBI build GRCh37 (UCSC hg19 assembly).
