# Supplementary material for: Multi-ethnic genome-wide association study identifies novel locus for type 2 diabetes susceptibility
Source: Eur J Hum Genet. 2016 May 18;24(8):1175–80. doi: 10.1038/ejhg.2016.17 (PMC4947384; doi:10.1038/ejhg.2016.17)
Supplement: Supplementary Information [file ejhg201617x1.pdf]

Multi-ethnic genome-wide association study identifies novel locus for type 2 diabetes susceptibility

Running title: Multi-ethnic genome-wide association analysis

James P Cook[1] and Andrew P Morris[1,2]

[1]Department of Biostatistics, University of Liverpool, Block F, Waterhouse Building, 1-5 Brownlow Street, Liverpool L69 3GA, UK. [2]Wellcome Trust Centre for Human Genetics, University of Oxford, Roosevelt Drive, Oxford OX3 7BN, UK.

SUPPLEMENTARY MATERIAL

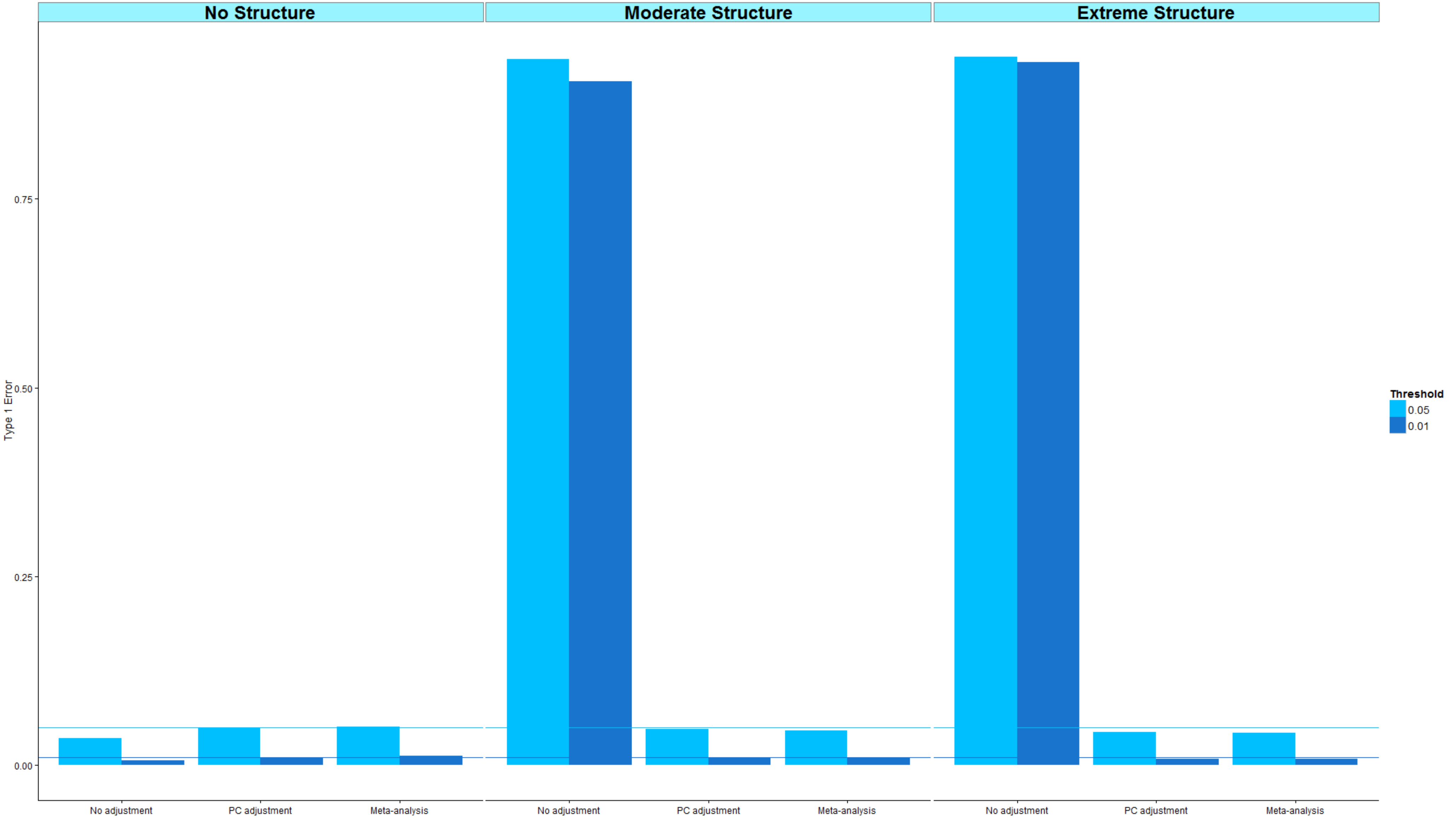

Supplementary Figure S1. Type I error rate for detecting association (at p<0.05 and p<0.01) of: (i) the logistic regression model, with and without adjustment for ten axes of genetic variation as covariates; and (ii) fixed-effects meta-analysis of summary statistics across populations (each adjusted for four population-specific axes of genetic variation) via inverse-variance weighting of effect sizes. The three panels correspond to extreme, moderate and no population structure (defined in Supplementary Table S2).

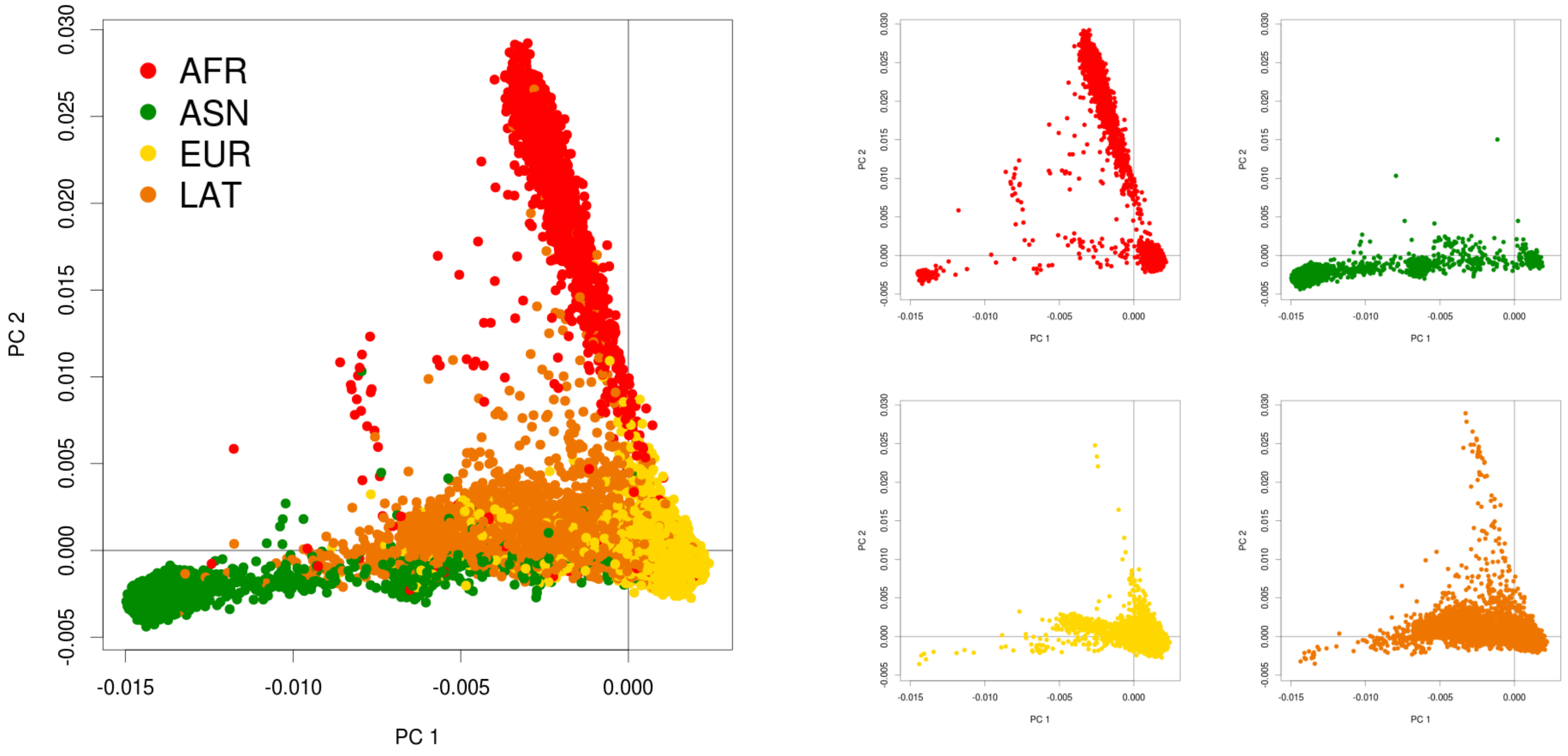

Supplementary Figure S2. Trans-ethnic population structure in 71,604 individuals from the GERA cohort obtained by plotting the first two axes of genetic variation (PC1 and PC2) from PCA (multi-dimensional scaling) of the genetic relatedness matrix. Each point corresponds to an individual, coloured according to the array used for their genotyping: AFR (African American); ASN (East Asian); EUR (non-Hispanic white); and LAT (Latino).

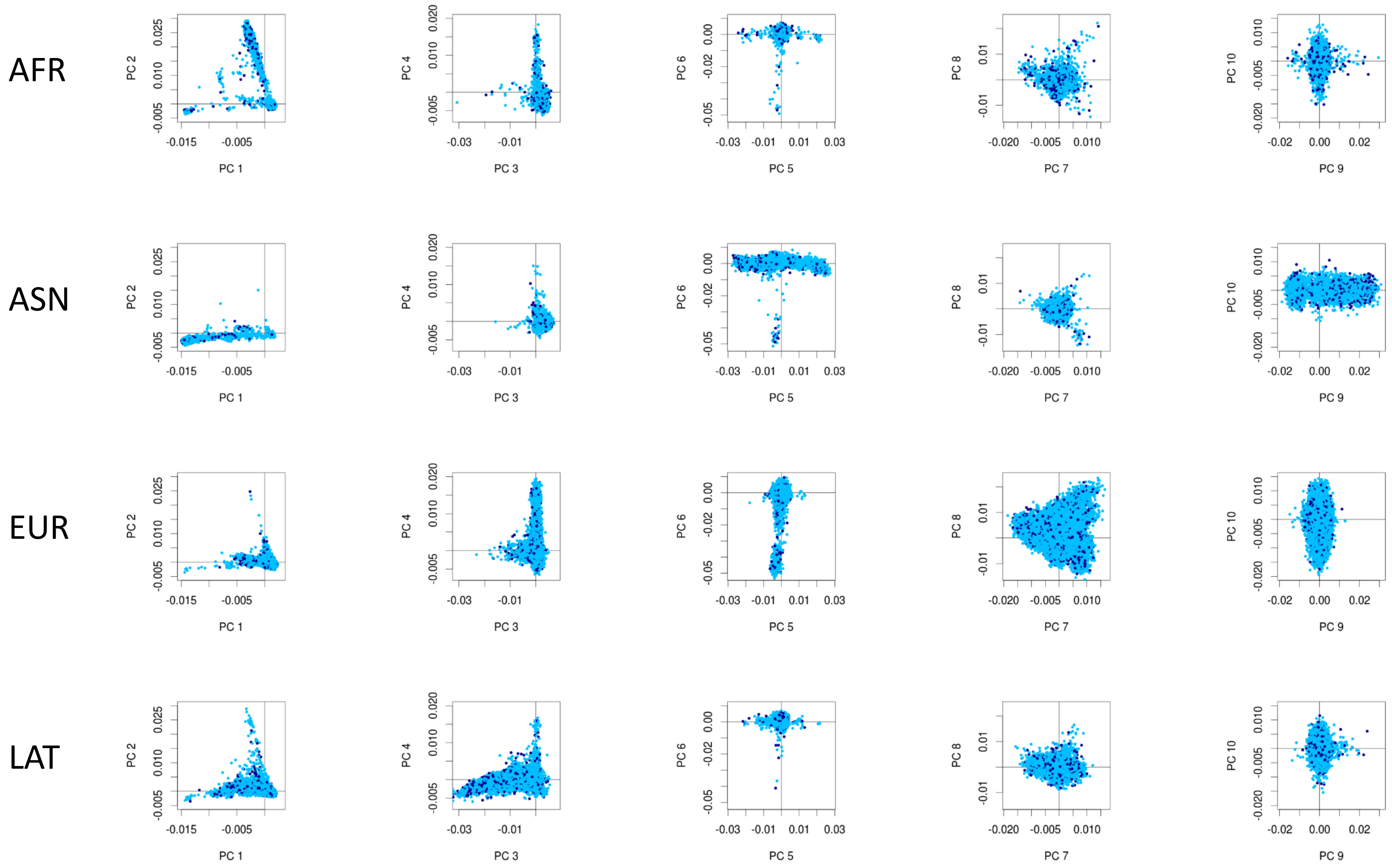

Supplementary Figure S3. Trans-ethnic population structure in 71,604 individuals from the GERA cohort obtained by plotting the first ten axes of genetic variation (PC1-PC10) from PCA (multi-dimensional scaling) of the genetic relatedness matrix, separately for each genotyping array: AFR (African American); ASN (East Asian); EUR (non-Hispanic white); and LAT (Latino). Each point corresponds to an individual, coloured according to T2D disease status: cases (dark blue) and controls (cyan).

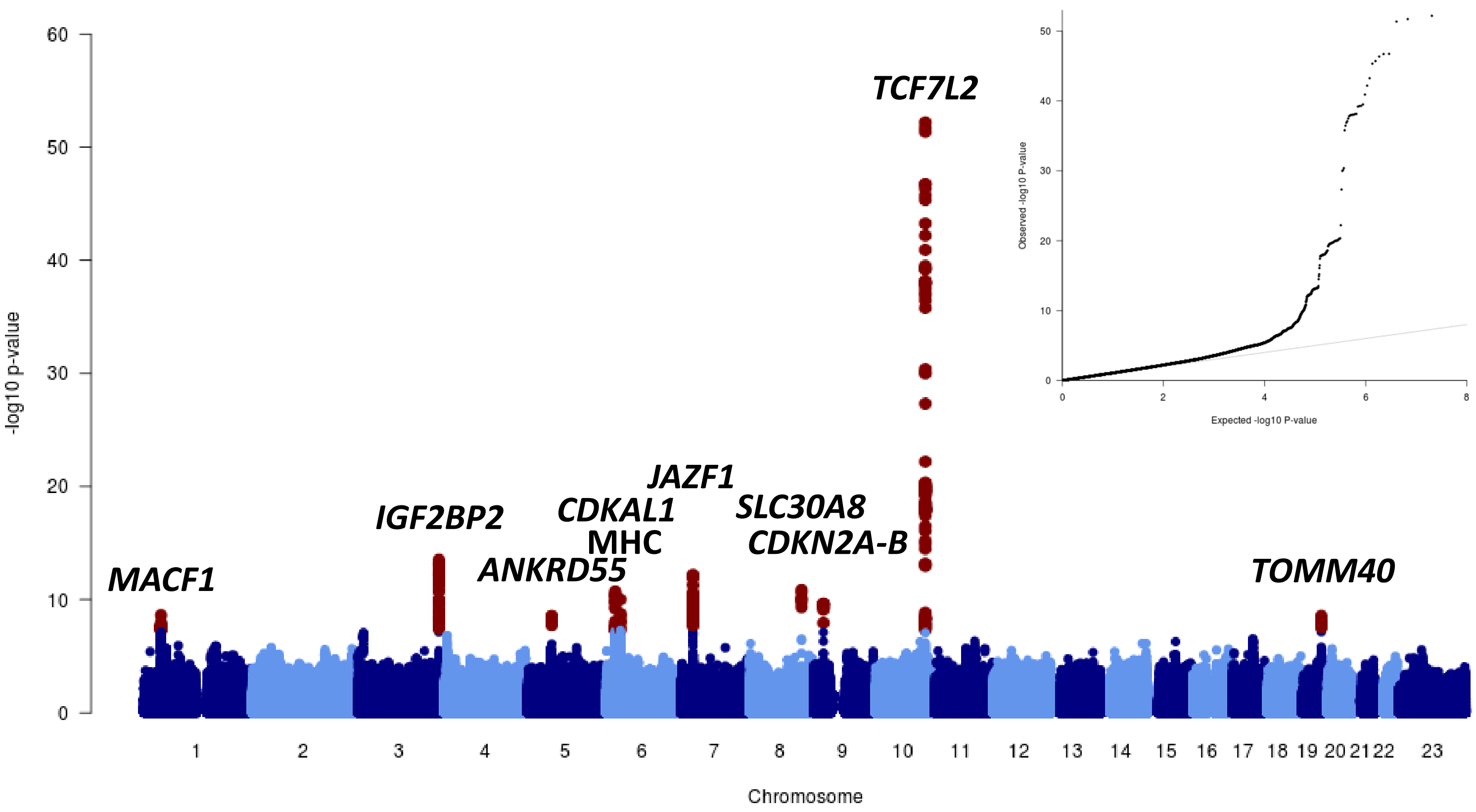

Supplementary Figure S4. Summary of genome-wide association of 10,143,997 imputed variants (MAF≥0.5%, info≥0.8) with susceptibility to T2D in 9,747 cases and 61,857 controls from the GERA cohort. In the Manhattan plot, each point corresponds to a variant, plotted according to physical position on the x-axis and $-\log_{10}$ *p*-value on the y-axis. Variants attaining genome-wide significance ($p<5\text{x}10^{-8}$) are highlighted in red. Locus names are given by the gene mapping closest to the lead SNP. In the quantile-quantile plot, each point corresponds to a variant, plotted according to the expected $-\log_{10}$ *p*-value on the x-axis, and the observed $-\log_{10}$ *p*-value of the y-axis.

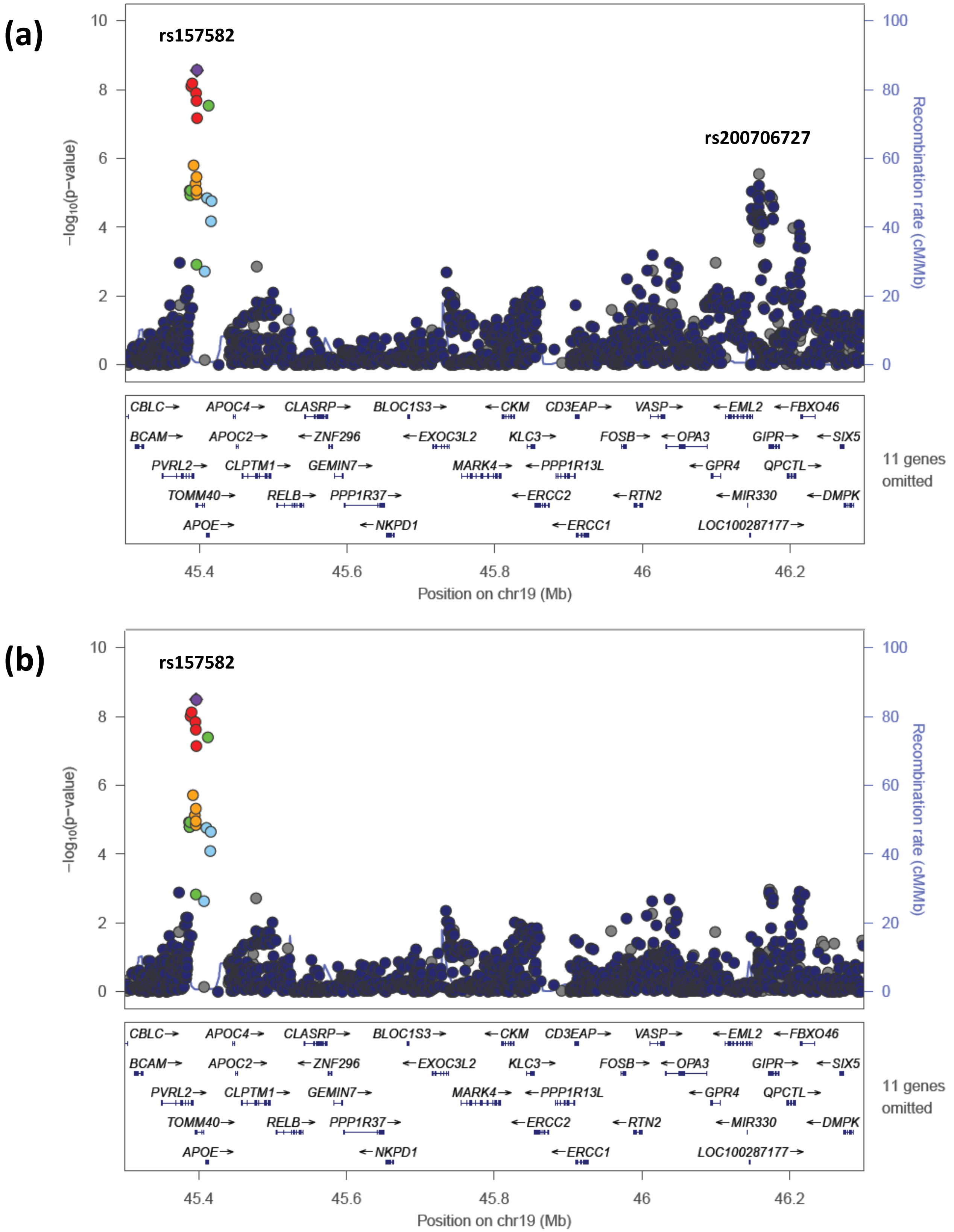

Supplementary Figure S5. Signal plot for T2D association signal mapping to *TOMM40-APOE* in 9,747 cases and 61,857 controls from the GERA cohort: (a) after adjustment for sex and nine axes of genetic variation as covariates; and (b) after additional adjustment for genotypes at the lead SNP (rs200706727) at the *GIPR* locus. Each point represents a SNP passing quality control in the association analysis, plotted with their *p*-value (on a $-\log_{10}$ scale) as a function of genomic position (NCBI build GRCh37, UCSC hg19 assembly). In each plot, the index variant is represented by the purple symbol. The colour coding of all other SNPs indicates LD with the index variant in European ancestry haplotypes from the 1000 Genomes Project reference panel: red $r^2 \geq 0.8$; gold $0.6 \leq r^2 < 0.8$; green $0.4 \leq r^2 < 0.6$; cyan $0.2 \leq r^2 < 0.4$; blue $r^2 < 0.2$; grey r^2 unknown. Recombination rates are estimated from Phase II HapMap and gene annotations are taken from the University of California Santa Cruz genome browser.

Supplementary Table S1. Models of association of the causal variant with a dichotomous phenotype across population groups.

Population	Ancestry	Population-specific allelic effect sizes (log-odds ratio)			
		No heterogeneity	African-specific effect	African vs others	East Asian vs European, South Asian and Hispanic
MKK	African	β	β	β	0
ASW	African	β	β	β	0
LWK	African	β	β	β	0
YRI	African	β	β	β	0
CHB/JPT	East Asian	β	0	$-\beta$	β
CHD	East Asian	β	0	$-\beta$	β
GIH	South Asian	β	0	$-\beta$	$-\beta$
MXL	Hispanic	β	0	$-\beta$	$-\beta$
CEU	European	β	0	$-\beta$	$-\beta$
TSI	European	β	0	$-\beta$	$-\beta$

Supplementary Table S2. Distribution of cases and controls in each population to induce confounding of the phenotype with ancestry group.

**Population**	**Ancestry**	**Number of cases/controls in each population**		
		**No structure**	**Moderate structure**	**Extreme structure**
MKK	African	1000/1000	500/1500	0/2000
ASW	African	1000/1000	500/1500	0/2000
LWK	African	1000/1000	500/1500	0/2000
YRI	African	1000/1000	500/1500	0/2000
CHB/JPT	East Asian	1000/1000	1000/1000	1000/1000
CHD	East Asian	1000/1000	1000/1000	1000/1000
GIH	South Asian	1000/1000	1500/500	2000/0
MXL	Hispanic	1000/1000	1500/500	2000/0
CEU	European	1000/1000	1500/500	2000/0
TSI	European	1000/1000	1500/500	2000/0
**Total**		**20000/20000**	**20000/20000**	**20000/20000**

Supplementary Table S3. Type I error rates for test of association with the phenotype: (i) with no adjustment for population structure; (ii) inclusion of ten axes of genetic variation from PCA as covariates to account for confounding; and (iii) fixed-effects meta-analysis of summary statistics across populations via inverse-variance weighting of effect sizes.

**Association test**	**Significance threshold**	**Type I error rate (SE)**		
		**No structure**	**Moderate structure**	**Extreme structure**
No adjustment for population structure	$p<0.05$	0.036 (0.006)	0.935 (0.008)	0.938 (0.008)
	$p<0.01$	0.006 (0.002)	0.906 (0.009)	0.931 (0.008)
Adjusting for ten axes of genetic variation	$p<0.05$	0.049 (0.007)	0.048 (0.007)	0.044 (0.006)
	$p<0.01$	0.010 (0.003)	0.010 (0.003)	0.008 (0.003)
Meta-analysis	$p<0.05$	0.051 (0.007)	0.046 (0.007)	0.043 (0.006)
	$p<0.01$	0.012 (0.003)	0.009 (0.003)	0.008 (0.003)

Supplementary Table S4. Summary of genotyping arrays utilised in the GERA cohort.

Genotyping array	cases/controls	Variants passing quality control	
		Scaffold	After imputation
African American (AFR)	746/2,825	667,685	19,067,441
East Asian (ASN)	743/3,992	618,313	11,114,505
Non-Hispanic white (EUR)	7,111/49,688	524,833	11,292,048
Latino (LAT)	1,147/5,352	642,679	12,577,765
Overlap		189,443	8,497,425

Supplementary Table S5. Axes of genetic variation from PCA (multidimensional scaling) of genetic the relatedness matrix that are associated with T2D susceptibility in 9,747 cases and 61,857 controls from the GERA cohort (where all summary statistics are adjusted for sex in the logistic regression model).

Axis of genetic variation	AFR genotyping array		ASN genotyping array		EUR genotyping array		LAT genotyping array		Trans-ethnic analysis		
	***p*-value**	**log-OR (SE)**	***p*-value**	**log-OR (SE)**	***p*-value**	**log-OR (SE)**	***p*-value**	**log-OR (SE)**	***p*-value**	**log-OR (SE)**	**Variance explained[a]**
PC1	0.038	-36.44 (17.60)	0.0017	-62.67 (19.94)	0.60	-15.24 (28.88)	0.99	-0.33 (28.36)	$<2.0 \times 10^{-16}$	-27.99 (2.78)	61.4%
PC2	9.8×10^{-7}	28.45 (5.81)	0.65	-34.71 (77.61)	0.31	21.82 (21.62)	0.82	-3.83 (17.27)	$<2.0 \times 10^{-16}$	32.83 (2.41)	17.7%
PC3	0.37	-25.65 (28.51)	0.18	-56.09 (42.02)	0.60	-7.32 (13.96)	0.0045	-22.28 (7.84)	$<2.0 \times 10^{-16}$	-24.64 (2.49)	7.5%
PC4	0.31	16.54 (16.27)	0.20	-57.50 (45.32)	0.00060	-15.61 (4.55)	0.59	-9.44 (17.59)	1.9×10^{-8}	-17.79 (3.16)	6.3%
PC5	0.46	10.46 (14.22)	0.039	-9.97 (4.84)	0.37	-8.12 (9.14)	0.081	-32.08 (18.39)	0.011	-6.64 (2.62)	2.0%
PC6	0.21	18.43 (14.65)	0.0025	-35.12 (11.60)	0.017	-9.19 (3.85)	0.26	-17.97 (16.10)	3.8×10^{-5}	-10.69 (2.60)	1.5%
PC7	0.41	-12.15 (14.84)	0.97	-0.78 (23.44)	1.1×10^{-7}	-17.86 (3.36)	0.69	4.58 (11.60)	3.6×10^{-8}	-16.47 (2.99)	1.4%
PC8	0.29	-15.91 (15.16)	0.47	-15.65 (21.66)	0.053	-6.27 (3.25)	0.099	-22.97 (13.91)	0.0048	-8.55 (3.03)	1.2%
PC9	0.19	19.31 (14.58)	1.9×10^{-12}	24.62 (3.50)	0.29	-6.71 (6.35)	0.51	-9.67 (14.63)	1.0×10^{-10}	17.69 (2.74)	1.0%

OR: odds ratio. SE: standard error.

AFR: African American. ASN: East Asian. EUR: non-Hispanic white. LAT: Latino.

[a]Relative phenotypic variance explained amongst first none axes of genetic variation.

Supplementary Table S6. Summary of interaction of the first two axes of genetic variation with genotypes at lead SNPs at T2D susceptibility loci in 9,747 cases and 61,857 controls from the GERA cohort.

Locus	Variant	Chr	Position[a] (bp)	HGVS ID	Alleles		RAF	Interaction with PC1 & PC2	Interaction with PC1		Interaction with PC2	
					Risk	Other		*p*-value	log-OR (SE)	*p*-value	log-OR (SE)	*p*-value
TCF7L2	rs34872471	10	114,754,071	NC_000010.10:g.114754071T>C	C	T	0.280	0.012	17.19 (8.55)	0.038	-5.32 (3.95)	0.18
IGF2BP2	rs11927381	3	185,508,591	NC_000003.11:g.185508591T>C	C	T	0.325	0.052	1.44 (4.26)	0.74	9.51 (3.97)	0.017
JAZF1	rs849134	7	28,196,222	NC_000007.13:g.28196222A>G	A	G	0.531	0.26	-7.61 (4.64)	0.10	-0.40 (3.92)	0.92
SLC30A8	rs13266634	8	118,184,783	NC_000008.10:g.118184783C>T	C	T	0.695	0.28	-0.31 (4.05)	0.94	-8.62 (5.42)	0.11
CDKAL1	rs7766070	6	20,686,573	NC_000006.11:g.20686573C>A	A	C	0.274	0.18	-6.91 (4.08)	0.091	-2.85 (4.12)	0.49
CDKN2A-B	rs10811661	9	22,134,094	NC_000009.11:g.22134094T>C	T	C	0.815	0.10	4.18 (4.23)	0.32	-12.60 (6.18)	0.042
MHC	rs9273401	6	32,627,129	NC_000006.11:g.32627129A>G	G	A	0.112	0.87	-0.76 (5.71)	0.89	-3.87 (7.57)	0.61
MACF1	rs3768321	1	40,035,928	NC_000001.10:g.40035928G>T	T	G	0.185	0.78	-1.36 (5.89)	0.82	-5.47 (8.45)	0.52
TOMM40-APOE	rs157582	19	45,396,219	NC_000019.9:g.45396219C>T	C	T	0.766	0.82	2.96 (5.28)	0.57	-0.81 (3.49)	0.82
ANKRD55	rs9687833	5	55,861,601	NC_000005.9:g.55861601G>A	A	G	0.200	0.63	3.10 (5.51)	0.57	3.32 (3.99)	0.40

Chr: chromosome. RAF: risk allele frequency. OR: odds ratio. SE: standard error.
[a]Position reported for NCBI build GRCh37 (UCSC hg19 assembly).

Supplementary Table S7. T2D association summary statistics from the multi-ethnic GERA cohort and the European ancestry DIAGRAMv3 meta-analysis for rs6857 (NC_000019.9:g.45392254C>T), aligned to risk allele C.

**Study**	**RAF**	***p*-value**	**OR (95% CI)**	**cases/controls**
GERA	0.844	$1.6x10^{-6}$	1.12 (1.07-1.17)	9,747/61,857
DIAGRAMv3	0.842	0.0025	1.11 (1.04-1.19)	12,171/56,862
**Combined**		**$7.4x10^{-9}$**	**1.12 (1.08-1.16)**	**21,918/118,719**

RAF: risk allele frequency. OR: odds ratio. CI: confidence interval.

Supplementary Table S8. T2D association summary statistics for the lead SNP at the *TOMM40-APOE* locus (rs157582, NC_000019.9:g.45396219C>T), after accounting for tag SNPs for *APOE* ε2 and ε4 alleles in conditional analyses, in 9,747 cases and 61,857 controls from the GERA cohort.

**Conditioning SNP(s)**			**T2D association**	
**ID**	**HGVS ID**	***APOE* allele**	***p*-value**	**OR (95% CI)[a]**
Unconditional	-	-	$8.1x10^{-9}$	1.12 (1.08-1.17)
rs429358	NC_000019.9:g.45411941T>C	ε4	0.0047	1.08 (1.02-1.14)
rs7412	NC_000019.9:g.45412079C>T	ε2	$1.2x10^{-8}$	1.12 (1.08-1.17)
rs429358<br>rs7412	NC_000019.9:g.45411941T>C<br>NC_000019.9:g.45412079C>T	ε4<br>ε2	0.016	1.07 (1.01-1.14)

OR: odds ratio. CI: confidence interval.
[a]OR aligned to risk allele C at rs157582.

Supplementary Table S9. T2D association summary statistics for the lead SNP at the TOMM40-APOE locus (rs157582, NC_000019.9:g.45396219C>T), stratified by age group, in 9,747 cases and 61,857 controls from the GERA cohort.

**Year of birth**	**cases/controls**	**OR (95% CI)[a]**	***p*-value**
Before 1939	4,262/17,790	1.15 (1.08-1.22)	$1.8x10^{-5}$
1939-1948	3,439/19,566	1.08 (1.01-1.15)	0.024
After 1948	2,046/24,501	1.10 (1.01-1.19)	0.021
Combined	9,747/61,857	1.12 (1.08-1.17)	$8.1x10^{-9}$

OR: odds ratio. CI: confidence interval.
[a]OR aligned to risk allele C at rs157582.
